# Supplementary material for: Iterative improvement in the automatic modular design of robot swarms
Source: PeerJ Comput Sci. 2020 Dec 7;6:e322. doi: 10.7717/peerj-cs.322 (PMC7924708; doi:10.7717/peerj-cs.322)
Supplement: Supplemental Information 3 [file peerj-cs-06-322-s003.zip › argos3/doc/api/standalone/a00625_source.html]

ARGoS: plugins/simulator/physics\_engines/pointmass3d/pointmass3d\_model.cpp Source File


- Main Page
- Related Pages
- Namespaces
- Classes
- Files

- File List
- File Members

# plugins/simulator/physics\_engines/pointmass3d/pointmass3d\_model.cpp

Go to the documentation of this file.

```
00001 #include "pointmass3d_model.h"
00002 #include <argos3/core/simulator/entity/embodied_entity.h>
00003 
00004 namespace argos {
00005 
00006    /****************************************/
00007    /****************************************/
00008 
00009    CPointMass3DModel::CPointMass3DModel(CPointMass3DEngine& c_engine,
00010                                         CEmbodiedEntity& c_entity) :
00011       CPhysicsModel(c_engine, c_entity),
00012       m_cPM3DEngine(c_engine) {
00013       /* Register the origin anchor update method */
00014       RegisterAnchorMethod(GetEmbodiedEntity().GetOriginAnchor(),
00015                            &CPointMass3DModel::UpdateOriginAnchor);
00016       /* Set initial position */
00017       m_cPosition = GetEmbodiedEntity().GetOriginAnchor().Position;
00018    }
00019    
00020    /****************************************/
00021    /****************************************/
00022 
00023    void CPointMass3DModel::Reset() {
00024       m_cPosition = GetEmbodiedEntity().GetOriginAnchor().Position;
00025       m_cVelocity = CVector3();
00026       m_cAcceleration = CVector3();
00027       CalculateBoundingBox();
00028    }
00029 
00030    /****************************************/
00031    /****************************************/
00032 
00033    void CPointMass3DModel::MoveTo(const CVector3& c_position,
00034                                   const CQuaternion& c_orientation) {
00035       m_cPosition = c_position;
00036       UpdateEntityStatus();
00037    }
00038 
00039    /****************************************/
00040    /****************************************/
00041 
00042    bool CPointMass3DModel::IsCollidingWithSomething() const {
00043       /* Go through other objects and check if the BB intersect */
00044       for(std::map<std::string, CPointMass3DModel*>::const_iterator it = GetPM3DEngine().GetPhysicsModels().begin();
00045           it != GetPM3DEngine().GetPhysicsModels().end(); ++it) {
00046          if((it->second != this) &&
00047             GetBoundingBox().Intersects(it->second->GetBoundingBox()))
00048             return true;
00049       }
00050       return false;
00051    }
00052 
00053    /****************************************/
00054    /****************************************/
00055 
00056    void CPointMass3DModel::UpdateOriginAnchor(SAnchor& s_anchor) {
00057       s_anchor.Position = m_cPosition;
00058    }
00059 
00060    /****************************************/
00061    /****************************************/
00062 
00063 }
```

---

Generated on 10 Jul 2018 for ARGoS by 
 1.6.1
